# Higher bone mineral density at six years of age in very preterm-born infants fed human milk compared to formula feeding. A secondary analysis of an RCT

**DOI:** 10.1007/s00431-024-05935-3

**Published:** 2025-01-30

**Authors:** Line H. Toftlund, Signe Sparre Beck-Nielsen, Lone Agertoft, Susanne Halken, Gitte Zachariassen

**Affiliations:** 1 Department of Neonatology, Children’s Department, Herlev Hospital, Borgmester Ib Juuls Vej 1, Herlev, 2730 Denmark; 2 Centre for Rare Diseases, Aarhus University Hospital and Institute for Clinical Research, Aarhus University, Aarhus, Denmark; 3 Department of Pediatrics, University Hospital of Southern Denmark, Aabenraa, Denmark; 4 Institute of Clinical Research, University of Southern Denmark, Odense, Denmark; 5 Hans Christian Andersen Children’s Hospital, Odense University Hospital, Odense, Denmark

**Keywords:** Very preterm infants, Post discharge nutrition, Bone mineral density

## Abstract

In very preterm-born infants, nutritional intake is important to reduce the risk of severe metabolic bone disease including the risk of a lower bone mineral density (BMD). The aim of this study was to evaluate bone mineral content (BMC) and BMD (measured as BMC per bone area (BA)) at six years of age in very preterm-born infants fed different diets post-discharge. Data on this topic so far is insufficient, and with this study we aim to supply more useful data. A prospective follow-up study of 281 children born very preterm (gestational age ≤ 32 + 0 weeks) and enrolled in a multicentre RCT on post-discharge nutrition. Infants fed human milk (HM) were randomised respectively to be fed unfortified HM (UHM) or fortified human milk (FHM) from hospital discharge to four months’ corrected age. Those not fed HM received a preterm formula (PF). At six years of age, BMD and BMC in all the children were established by means of a dual-energy X-ray absorptiometry (DXA) scan (Lunar Prodigy) and adjusted for sex, age, and anthropometrics. A total of 192 very preterm-born children (59 fed UHM, 67 FHM and 66 PF) had a DXA scan performed at median 6 (5.8—8.3) years of age. No significant difference was found comparing UHM and FHM according to height, weight, BA, BMC, and BMD at six years of age. However, a multiple regression analysis showed significantly improved BMD in breastfed children compared to PF-fed children.

*Conclusions*: Fortified compared to non-fortified human milk post-discharge did not have an impact on BMD at 6 years of age in very preterm-born infants. Breastfed children demonstrated higher BMD than formula-fed children.

**Table:** 

**What is Known:**
• *Adequate nutritional intake is important to improve growth and to reduce the risk of severe bone disease in very preterm born infants.*
•* Bone mineralization is attained later in preterm born infants compared to term born infants.*
**What is New:**
• *Feeding human milk with fortification compared to non-fortified human milk did not improve bone mineral density in children born very preterm in this follow-up study at six years of age.*
• *Feeding human milk compared to formula was associated with increased BMD at six years of age among very preterm born infants.*

## Introduction

With preterm birth, the considerable nutrient flow from the placenta is abruptly terminated. Since the preterm infant has very low skeletal mineral stores at birth, optimal supplementation of nutrients and minerals is essential in order to achieve the most optimal bone formation and mineralization after birth, and to reduce the risk of severe metabolic bone disease in the preterm infant [1–3]. In accordance with our knowledge of the excessive growth and mineral accretion during a normal third trimester, there is a considerable need for calcium and phosphate uptake to complete bone mineralization in infants born very preterm [4]. This is challenged by the often compromised and immature gut. Inadequate bone mineralization at any time during infancy/childhood implies an increased risk of not achieving the genetically determined optimal peak bone mass in adulthood. Studies suggest that achievement of an optimal peak bone mass is an important predictor for the risk of osteoporosis and thus osteoporotic fractures later in life. Optimizing bone mineralization in the preterm-born infant is therefore essential [4, 5]. Research in ensuring a sufficient and balanced nutrient and mineral supply from birth to discharge has been performed and recommendations published [6], whereas data regarding the potential outcome in very preterm infants fed different diets post-discharge with respect to bone mineral density (BMD) is sparse [7]; thus, studies in this field will be an important contribution to this knowledge. We therefore found it important to explore the effect(s) of different post-discharge nutritional regimens on future BMD.

The aim of the present study was to investigate whether fortifier added to the mother’s own milk post-discharge would lead to improved bone mineral content (BMC) and/or density (BMD) at six years of age in very preterm-born infants. We compared infants fed fortified human milk (FHM), unfortified human milk (UHM), or a preterm formula (PF) post-discharge, respectively.

## Methods

A total of 320 very preterm-born (gestational age (GA) ≤ 32 + 0 weeks) children who participated in a prospective, randomized, controlled multicentre trial (RCT) on post-discharge nutrition and growth observation until 1 year of life [8] were invited to participate in a follow-up study at age six years. In the original RCT, the infants were born from 2004 through 2008. Follow-up was conducted from 2010 to 2015. Results on growth and metabolic outcome at 6 years of age have previously been published [9–11]. Data on prenatal steroids for the mother and postnatal steroids for the infants has not been registered. No infants with bronchopulmonary dysplasia, necrotising enterocolitis treated with surgery, or severe intra-ventricular haemorrhage (IVH 3–4) participated in the original RCT. None of the infants in the study were diagnosed with rickets, osteopenia or fractures during hospitalization.

Nutrition during hospitalization until full enteral feeding was established around day 10–14, was supplemented with parenteral nutrition (PN). PN was routinely used in infants with a GA below 28 weeks, and on an individual basis in preterm-born infants with a GA between 28–32 weeks. Unfortunately, data on PN during hospitalization is unknown. From day 10–14 after birth, HM was fortified with a Human Milk Fortifier for all breastfed infants until discharge. All infants received vitamin D supplementation with 10 μg regardless of mode of feeding from their second week of life and until 2 years of age, as recommended at that time by the Danish Health Authority.

At discharge, three groups were established. Breastfed participants were randomised to be fed either unfortified human milk (UHM) or fortified HM (FHM). Those not breastfed received a preterm formula. Details on randomisation and fortification are described elsewhere [8].

At follow-up at six years of age, a whole-body dual-enerχgy X-ray absorptiometry (DXA) scan was performed in each of the children with the GE Lunar Prodigy (GE Medical Systems, Madison, WI), ENCORE software (Version 15. Prodigy; Lunar Corp, Madison, WI). The DXA scans were conducted between 2010 and 2015 at Odense University Hospital, Denmark.

The DXA scans provided information on bone area (BA), BMC and BMD with total body less head (TBLH) values on both BMC and BMD. The latter were used for further analyses as advised by the International Society for Densitometry [12]. Further Z-scores on BMD were calculated using the reference for Caucasian children (US, Australia and Northern Europe) provided by GE Lunar Prodigy. The BMD value obtained by DXA is not a true density, as it is derived from a projected two-dimensional area of a three-dimensional bone structure. Thus, the BMD value obtained is an area BMD (gram/cm^2^) and not a volumetric BMD (gram/cm^3^). As the depth of the projected bone is not determined, and size artifact arises, smaller bones appear to have a lower area BMD than larger bones despite equal volume BMD [13]. To correct for this confounder, weight and height Z-scores (equal to SD scores) registered at the time of DXA were included in the regression model when comparing BMD values of the 3 feeding groups.

The reproducibility of the GE Lunar Prodigy has previously been evaluated with a margin of 0,74% Coefficient of Variation (CV) for BMC, 0.75% CV for BMD, and 1.97% CV for area by standard scan mode in children with a mean age of 11.4 years (5–17 years) [14]. The radiation dose level was below 0.50 µSv (effective dose). DXA scans were conducted with the children wearing only socks and a T-shirt and/or underwear. Anthropometric data: height was measured to the nearest 0.1 cm using a portable stadiometer (SECA 214). Weight was measured to the nearest 0.1 kg on an electronic scale (SECA 861) (Seca Corporation, Hannover). Height and weight measurements were conducted with the children being barefoot, wearing only a T-shirt and/or underwear.

### Statistical analyses

Data was analysed using Stata 12 (Stata Corp, College Station, TX). Student´s t-test was used for comparison of continuous and χ^2^-test for categorical variables. A Skewness-Kurtosis test was used to test if the data was normally distributed. The data for GA at discharge and corrected age at time of DXA was not normally distributed, and these two categories are reported as medians. Lean body mass (LBM), FM, BMC and BA were normally distributed by log transformation. BMD and BMD Z-scores were normally distributed.

Nutrition groups were compared as UHM vs FHM, FHM vs PF, UHM vs PF, human milk (HM) = (UHM + FHM) vs PF, and UHM vs protein supplementation (FHM + PF). A multiple regression analysis was used to evaluate the relationship between post-discharge nutrition, BA, BMC, BMD and BMD Z-score at six years of age in very preterm-born infants. BA, BMC, BMD or BMD Z-score were the dependent variables. Nutrition group, gender, small for gestational age at birth (SGA), weight growth (Z-score difference) from postmenstrual age of 34 weeks and until corrected age of 2 months [11], LBM, height *Z*-score, and weight *Z*-score at time of DXA, which are all suggested as having an effect on bone variables during childhood, were the independent variables [15]*.* To compare height and weight measurements according to gender and time, *Z-*scores were calculated as the difference between the actual variable, e.g. weight, and the expected reference weight divided with 1 SD (e.g. [birth weight—reference birth weight]/1 SD) for each gender. Small for gestational age (SGA) was defined as a birth weight *Z-*score below or equal to −2 SD. *Z-*scores at birth were calculated according to a reference for each gender[16], and anthropometrics (weight and height) obtained at 5–8 years of age were expressed in Z-scores, adjusting for sex and age according to Danish reference data [17]. *P-*values < 0.05 were considered significant.

## Results

**Fig. 1:**
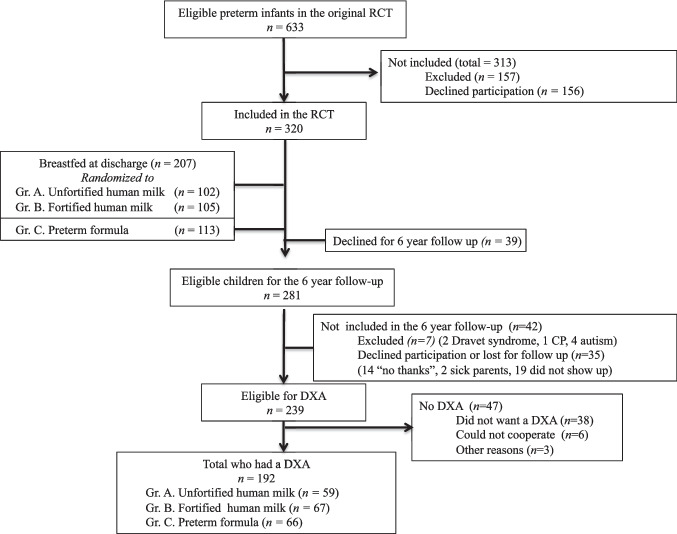
Flowchart; patient inclusion, exclusion and drop-outs

A total of 192 children out of the original 320 participants had a DXA scan performed at 6 (5.8—8.3) years of age. The number of dropouts and excluded participants was 43 in UHM, 38 in FHM, and 47 in PF groups, respectively (Fig. 1). Of the remaining 192 children, all completed a full DXA, leaving us with no missing data regarding DXA. Results on basic characteristics are shown in (Table 1).

**Table 1:** Baseline characteristics

	UHM	FHM	(UHM+FHM) HM	PF	(FHM+PF) Protein	UHM vs FHM *t*-test	HM vs PF *t*-test	UHM vs Pro. *t*-test
Children DXA *n*	59	67	126	66	133			
SGA at birth *n*	11	13	24	17	30	0.35	1.00	0.70
Boys *n*	29	30	59	41	71	0.05*	0.72	0.64
Multiple birth *n*	12	28	40	30	58	0.08	0.01*	0.002*
GA								
Median	204.0	213.0	209.5	207.0	212.0			
Min	169.0	171.0	169.0	184.0	171.0			
Max	224.0	224.0	224.0	222.0	224.0			
Birth weight (g)								
Median	1221.0	1330.0	1264.5	1282.5	1294.0			
Min	548.0	690.0	548.0	612.0	612.0			
Max	2255.0	2024.0	2255.0	2140.0	2140.0			
Age at discharge (days)								
Median	262.0	261.0	261.5	257.0	259.0			
Min	243.0	243.0	243.0	245.0	243.0			
Max	319.0	297.0	319.0	286.0	297.0			
Age at DXA (years)								
Median	6.3	6.3	6.3	6.2	6.3			
Min	5.8	5.8	5.8	5.8	5.8			
Max	8.3	7.7	8.3	7.2	7.7			
Weight at DXA (kg)								
Median	21.5	20.8	21.1	21.4	21.0			
Min	16.0	13.9	13.9	16.8	13.9			
Max	35.1	33.8	35.1	40.0	40.0			
Weight growth (Z-score dif. from GA 34 to 2 months CA)								
Mean	0.79	0.96	0.88	1.31	1.13	0.28	0.002*	0.01*
SD	0.87	0.88	0.87	0.91	0.91			
Weight z-score DXA								
Mean	–0.16	–0.40	–0.29	–0.09	–0.24	0.22	0.27	0.62
SD	1.06	1.14	1.10	1.24	1.20			
Height at DXA (cm)								
Mean	120.61	119.56	120.05	118.97	119.27	0.28	0.18	0.12
SD	5.56	5.16	5.35	5.35	5.19			
Height z-score DXA								
Mean	0.11	0.01	0.06	–0.10	–0.04	0.58	0.31	0.32
SD	0.99	1.10	1.04	1.00	1.05			
LBM								
Mean	9.75	9.71	9.73	9.73	9.72	0.10	0.79	0.12
SD	0.14	0.13	0.13	0.13	0.12			
FM								
Mean	8.03	8.02	8.02	8.14	8.08	0.90	0.15	0.55
SD	0.53	0.52	0.52	0.57	0.54			

*GA*, gestational age and *BW*, birth weight, no significant difference comparing groups. *CA*, corrected age; *LBM*, lean body mass; *FM*, fat mass; *n*, numbers

At six years follow-up, no significant difference was found when comparing the FHM group with the UHM group regarding bone variables. There were significantly more multiple births in the PF-group than HM-group, and rapid growth occurred more often in PF compared to UHM.

The multiple regression analysis correcting for variables such as gender, BW, SGA, growth from 34 weeks to 2 months CA, weight, and height at time of DXA and LBM showed a significantly higher BMC, BMD and BMD *Z*-score in breastfed children (UHM + FHM) compared to formula-fed children (PF). There was no significant difference between UHM and FHM on any bone outcomes. Nor did we find any significant difference when pooling those who received extra protein versus those who were fed solely breastmilk (Table 2).

**Table 2:** Results on bone outcomes at 6 years of age

			(UHM + FHM) HM		(FHM + PF) Protein	UHM vs FHM t-test	HM vs PF t-test	UHM vs Pro t-test	UHM vs FHM Adjusted	HM vs PF Adjusted	UHM vs Pro Adjusted
	UHM	FHM		PF							
**Children DXA** *n*	59	67	126	66	133						
**BMC (g)**											
Mean	6.16	6.14	6.15	6.11	6.13	0.53	0.23	0.27			
SD	0.21	0.18	0.20	0.22	0.20						
Regression:											
Beta									0.02	0.04	−0.00
CI									(−0.01;0.06)	(−0.07;−0.01)	(−0.03;0.03)
*P*-value									0.21	0.009*	0.99
**Bone area (cm2)**											
Mean	6.60	6.58	6.59	6.57	6.58	0.48	0.43	0.33			
SD	0.15	0.14	0.15	0.16	0.15						
Regression:											
Beta									0.02	−0.02	0.01
CI									(−0.01;0.04)	(−0.04;0.00)	(−0.02;0.03)
*P*-value									0.17	0.10	0.62
**BMD (g/cm2)**											
Mean	0.65	0.64	0.65	0.63	0.64	0.65	0.06	0.19			
SD	0.04	0.04	0.04	0.04	0.04						
Regression:											
Beta									0.00	−0.02	0.00
CI									(−0.01;0.01)	(−0.03;−0.01)	(−0.01;0.01)
*P*-value									0.46	0.001*	0.47
**BMD z-score**											
Mean	−0.15	−0.17	−0.16	−0.32	−0.25	0.86	0.22	0.44			
SD	0.79	0.75	0.77	0.89	0.82						
Regression:											
Beta									0.09	−0.28	−0.04
CI									(−0.12;0.31)	(−0.48;0.09)	(−0.24;0.15)
*P*-value									0.39	0.004*	0.66
**Below −1 SD** *n*	9	7	16	17	24	0.03*	0.44	0.68			
**Below −2 SD** *n*	2	0	2	1	1	1.00	0.22	0.22			

Regression analysis adjusted for nutrition, gender, SGA, weight and height z-scores at 6 years of age, growth from week 34 to 2 months CA, and LBM

*SD* standard deviation, *CI* confidence interval

^*^Significant when *p*-value < 0.05

## Discussion

This is one of the first published RCT studies investigating the potential effect of different post-discharge nutritional regimens on BMD in 6-year-old children born very preterm. We did not find a difference in bone variables comparing the two human milk-fed groups, but we found a significant difference when comparing human milk feeding with formula feeding. In our previous studies, we found early rapid growth (weight growth > 1 SD from 34 weeks to 2 months CA) to have a potential influence on metabolic outcomes at six years of age [9, 10]. Early rapid growth did not seem to influence bone variables. A similar study, with a cohort of very preterm infants born from 2003 to 2006, did a follow up at 8 years of age. In this study, infants were randomised to a preterm or a term formula post-discharge, and the third group was a human milk-fed group. At six years of age, researchers did not find any difference in metabolic outcomes or bone variables [18].

Several studies have investigated the impact of feeding during the hospitalization period on BMC in preterm infants. Recently Einloft et al*.* performed a study in which 83 preterm infants with a birth weight of less than 1500 g were randomized to receive HM with or without supplementation with FM85®. DXA was performed at study entry and when the infant reached 2000 g, shortly before discharge. Whole body BMC was established in 38 infants, and was found to be significantly higher at the end of the study in infants fed fortified HM. Also, the level of alkaline phosphatase (ALP) was significantly higher among infants fed HM without supplementation [19]. Higher BMC and lover ALP in the fortified group indicates a need for extra protein. ALP’s applicability in distinguishing the presence of metabolic bone disease in preterm infants is questionable [20]. It is reasonable to assume that the lower BMC and higher ALP in the HM group is caused by a higher bone turnover aiming to maintain the serum values of minerals within the normal range due to insufficient enteral supply. Unfortunately, measurements of ALP were not performed in our study, thereby limiting the evaluation of possible differences in osteoblastic activity according to different post-discharge diets.

In a randomized double blinded study by Koo et al., 89 preterm infants with a GA of 24–34 weeks were randomized to receive nutrient-enriched formula with a higher energy density, higher protein, calcium, and phosphate content compared to a standard formula for term infants. The formulas were given for one year after hospital discharge, and 67 infants completed the study. At 2-, 4-, 6-, and 12-months post-discharge, DXA scans were obtained. Infants fed term formula surprisingly turned out to have higher weight and length Z-scores compared to infants fed the enriched formula. This increase in body size was also reflected in a higher total body BMC in the children fed term formula. The authors did not measure area BMD or volumetric BMD, and the recommended setting TBLH was not used, most likely confounding the results [21]. Figueras-Aloy et al*.* reported a higher BMD in preterm infants when fed fortified human milk compared with a preterm formula. The study was a prospective study including 336 preterm infants with a GA ≤ 31 weeks and birth weight ≤ 1500 g. DXA was performed before discharge with analysis of the BMD value of the lumbar spine L1-L4. Meanwhile, precaution must be applied in the interpretation of the BMD of the lumbar spine in only a few months old preterm infants, as the vertebrae are largely cartilaginous at birth, which may indeed challenge the edge detection of the vertebrae [22].

A takeaway from these studies is that we don’t need to enrich the formula but continue to include HM as a main component in nutrition for very preterm infants. Further, it is important to choose the most frequently reported data (BMD/BMC) and thereby be able to compare studies. Often BMD is used, as this data gives the bone mineral content per square root of the bone, making it a more precise measure of the bone, regardless of the height of the person having a DXA scan.

Bishop et al*.* published data on a subgroup of 54 preterm children randomized to banked HM without fortification versus PF as a supplement to maternal HM. By single photon absorptiometry (SPA), and when adjusting for body size, the authors found a strong positive correlation between BMC at the age of five years and a high percentage of HM (maternal milk and banked HM) compared to HM and supplementation with formula [23]. This supports our finding of a higher BMD at six years of age in the group fed HM versus formula feeding, as our results also suggest that HM is important for bone growth.

Fewtrell et al*.* performed a follow-up study of the same preterm-born children with DXA scans at ages 8–12 years, and this revealed no differences in bone density related to different diets during hospitalization in infancy [24]. In a later study in 20-year-old adults from a preterm cohort by Fewtrell et al*.*, DXA scans showed a higher whole-body BMC associated with HM feeding compared to formula feeding in preterm-born infants during the first month of life [25]. However, this effect disappeared when correcting for whole body bone area, emphasizing the importance of appropriate size adjustments when interpreting DXA results, with children as well adults. As the uncorrected BMC was higher in the group fed HM, the authors conclude that feeding HM during the first months of preterm infants’ lives may contribute to larger bones with a proportional increase in mineral mass at age 20 years [25]. Fewtrell et al*.* concluded that the increased whole-body bone mass may reflect non-nutritive factors in the mother’s own “low nutrient milk” compared to a very high nutrient content when formula fed [26]. In our study, there was no difference in BMD at six years of age when comparing children fed low nutrient diet versus high nutrient diet (group UHM vs. FHM + PF). We therefore agree that other substances than those added by supplementation to breast milk might be responsible for the achievement of a higher bone density at age six years.

Interestingly, a study of formula fed versus breastfed term born children showed no difference in bone mass by DXA, when examined at the age of 10 years [27]. We have not found other studies showing an effect on bone mineralization among term-born infants fed different diets, but our data indicates a favourable effect of breast versus formula feeding with respect to BMD in children born very preterm. Preterm-born infants are at risk of poor bone mineralization in early life due to mineral deficit at birth and insufficient nutrition while treated at the NICU. However, nutritional intake has been improved in many NICU’s according to recent recommendations from e.g. ESPGHAN on both parenteral and enteral intakes including calcium and vitamin D [6]. In our RCT, all infants received HM with fortification or PF and vitamin D and phosphate during hospitalization as recommended. The results of the present follow-up study primarily illustrate the influence of different post-discharge nutrition until four months, and not solely the nutrition during hospitalization.

Lucas et al*.* randomized 229 premature infants (GA < 37 weeks with birth weight < 1750 g) to an enriched post-discharge formula or a standard term formula from discharge until nine months corrected age. A reference group of 65 preterm infants were breastfed for at least six weeks post-term. The authors found breastfed infants to be shorter and lighter, but raised the question in 2001 of whether infants breastfed post-discharge need nutritional supplementation [28]. We did not find any difference in anthropometric data at four months of CA [8] or at six years of age associated with the fortification of human milk post-discharge [11]. The evidence in favour of advising fortification of breast milk has been addressed in a recent Cochrane review, concluding that the data available is limited and does not provide strong evidence that supplementing preterm infants with fortification is superior to HM, but provides low-quality evidence of increased growth rates (weight, length, and head circumference) before hospital discharge. Very limited data was available for outcomes beyond infancy [29], and the results seem to be in accordance with our results, showing no effect on growth or bones at six years when infants were fed fortified HM versus HM post-discharge.

Strengths and limitations: Our study was conducted in a prospective design, randomized to supplementation with or without fortification when breastfed post-discharge. It is known that severe premature birth-related co-morbidities and medications could possibly influence bone health later in life in very preterm infants [30]. Medication including details on PN and diseases known to affect bone mineralization led to exclusion from the study. The dose of added fortifier to mother’s milk was relatively low and was not increased during the trial period. The measured values of blood urea nitrogen and growth were not significantly different between the fortified and unfortified groups [8], which also justifies combining the human milk-fed groups for comparison with preterm formula. The differences in level of physical activity, calcium intake and/or vitamin D status during infancy and early childhood may have influenced the BMD at age six years, but this potential effect is expected to be of equal impact in all three groups.

Future studies on the effects of early nutrition on BMD in preterm-born children need to be reported in follow-up studies, since data is still sparse.

## Conclusion

Nutrient enrichment of mothers’ own milk did not improve BMD, but feeding HM with or without fortification compared to formula feeding post-discharge was associated with increased BMD at six years of age among very preterm-born infants. With respect to the objective of achieving the optimal peak bone mass, feeding HM (with or without fortification) appears to be superior to formula feeding post-discharge in very preterm-born infants.

## Data Availability

No datasets were generated or analysed during the current study.

## Abbreviations and definitions

AGA: Appropriate for gestational age (-2 SDS < BW z-score < 2 SDS)
BA: Bone area
BMC: Bone mineral content
BMD: Bone mineral density
BW: Birth weight in gram
CA: Corrected age (weeks, months, or year after birth)
CI: Confidence interval
DXA: Dual-energy X-ray absorptiometry
FHM: Fortified Human Milk
GA: Gestational age (weeks and days)
HM: Human milk
HMF: Human milk fortifier
LBM: Lean body mass
NICU: Neonatal Intensive Care Unit
PMA: Postmenstrual age (weeks and/or days since last menstrual period before birth)
PF: Preterm formula
Protein (Pro.): A combined group of both FHM and PF(children, who received extra protein)
SGA: Small for gestational age (BW z-score ≤ -2 SDS according to a reference)
SD: Standard deviation
TBLH: Total body less head
UHM: Unfortified Human Milk
Z-score: Also referred to as standard deviation score (SDS)
