# Correction to: Higher bone mineral density at six years of age in very preterm-born infants fed human milk compared to formula feeding. A secondary analysis of an RCT

**DOI:** 10.1007/s00431-025-06045-4

**Published:** 2025-03-05

**Authors:** Line H. Toftlund, Signe Sparre Beck-Nielsen, Lone Agertoft, Susanne Halken, Gitte Zachariassen

**Affiliations:** 1 Department of Neonatology, Children’S Department, Herlev Hospital, Borgmester Ib Juuls Vej 1, 2730 Herlev, Denmark; 2 Centre for Rare Diseases, Aarhus University Hospital and Institute for Clinical Research, Aarhus University, Aarhus, Denmark; 3 Department of Pediatrics, University Hospital of Southern Denmark, Aabenraa, Denmark; 4 Institute of Clinical Research, University of Southern Denmark, Odense, Denmark; 5 Hans Christian Andersen Children’s Hospital, Odense University Hospital, Odense, Denmark

**Correction to: European Journal of Pediatrics (2025) 184:160**

10.1007/s00431-024-05935-3

In the originally published article entitled “Higher bone mineral density at six years of age in very preterm-born infants fed human milk compared to formula feeding. A secondary analysis of an RCT”, Table 1 is very unregular and is difficult to read, unfortunately.

**Published version:**

**Table 1 **Baseline characteristics

**Table:** 

	UHM			FHM	(UHM + FHM) HM		PF	(FHM + PF) Protein	UHM vs FHM t-test *p*-value	HM vs PF t-test *p*-value	UHM vs Pro t-test *p*-value
**Children DXA** *n*	59			67	126		66	133			
**SGA at birth** *n*	11			13	24		17	30	0.35	1.00	0.70
**Boys** *n*	29			30	59		41	71	0.05*	0.72	0.64
**Multiple birth** *n*	12			28	40		30	58	0.08	0.01*	0.002*
**GA** *(days)*											
Median 204	204			213	209		207	212			
Min	169			171	169		184	171			
Max	224			224	224		222	224			
**Birth weight** (g)											
Median	1221			1330	1265		1282	1294			
Min	548			690	548		612	612			
Max	2255			2024	2255		2140	2140			
**Age at discharge** (days)											
Median	262			261	262		257	259			
Min	243			243	243		245	243			
Max	319			297	319		286	297			
**Age at DXA** (years)											
Median	6.3			6.3	6.3		6.2	6.3			
Min	5.8			5.8	5.8		5.8	5.8			
Max	8.3			7.7	8.3		7.2	7.7			
**Weight at DXA** (kg)											
Median	21.5		20.8			21.1	21.4	21.0			
Min	16.0		13.9			13.9	16.8	13.9			
Max	35.1		33.8			35.1	40.0	40.0			
**Weight growth** (Z-score dif. from GA 34 to 2 months CA)											
Mean	0.79	0.96				0.88	1.31	1.13	0.28	0.002*	0.01*
SD	0.87	0.88				0.87	0.91	0.91			
**Weight z-score DXA**											
Mean	− 0.16	− 0.40				− 0.29	− 0.09	− 0.24	0.22	0.27	0.62
SD	1.06	1.14				1.10	1.24	1.20			
**Height at DXA** (cm)											
Mean	120.61	119.56				120.05	118.97	119.27	0.28	0.18	0.12
SD	5.56	5.16				5.35	5.23	5.19			
**Height z-score DXA**											
Mean	0.11	0.01				0.06	− 0.10	− 0.04	0.58	0.31	0.32
SD	0.99	1.10				1.04	1.00	1.05			
**LBM**											
Mean	9.75	9.71				9.73	9.73	9.72	0.10	0.79	0.12
SD	0.14	0.13				0.13	0.13	0.12			
**FM**											
Mean	8.03	8.02				8.02	8.14	8.08	0.90	0.15	0.55
SD	0.53	0.52				0.52	0.57	0.54			

*GA* gestational age, and *BW* birth weight, no significant difference comparing groups, *SGA* small for gestational age, *CA* corrected age, *LBM* lean body mass, *FM* fat mass, *n* numbers, *UHM *unfortified human milk, *FHM* fortified human milk, *HM* human milk (UHM + FHM), *PF* preterm formula, *Pro* protein (PF + FHM), *dif* difference

**Corrected form:**

**Table 1** Baseline characteristics

**Table:** 

	UHM	FHM	(UHM + FHM) HM	PF	(FHM + PF) Protein	UHM vs FHM t-test	HM vs PF t-test	UHM vs Pro. t-test
Children DXA *n*	59	67	126	66	133			
SGA at birth *n*	11	13	24	17	30	0.35	1.00	0.70
Boys *n*	29	30	59	41	71	0.05*	0.72	0.64
Multiple birth *n*	12	28	40	30	58	0.08	0.01*	0.002*
GA								
Median	204.0	213.0	209.5	207.0	212.0			
Min	169.0	171.0	169.0	184.0	171.0			
Max	224.0	224.0	224.0	222.0	224.0			
Birth weight (g)								
Median	1221.0	1330.0	1264.5	1282.5	1294.0			
Min	548.0	690.0	548.0	612.0	612.0			
Max	2255.0	2024.0	2255.0	2140.0	2140.0			
Age at discharge (days)								
Median	262.0	261.0	261.5	257.0	259.0			
Min	243.0	243.0	243.0	245.0	243.0			
Max	319.0	297.0	319.0	286.0	297.0			
Age at DXA (years)								
Median	6.3	6.3	6.3	6.2	6.3			
Min	5.8	5.8	5.8	5.8	5.8			
Max	8.3	7.7	8.3	7.2	7.7			
Weight at DXA (kg)								
Median	21.5	20.8	21.1	21.4	21.0			
Min	16.0	13.9	13.9	16.8	13.9			
Max	35.1	33.8	35.1	40.0	40.0			
Weight growth (Z-score dif. from GA 34 to 2 months CA)								
Mean	0.79	0.96	0.88	1.31	1.13	0.28	0.002*	0.01*
SD	0.87	0.88	0.87	0.91	0.91			
Weight z-score DXA								
Mean	−0.16	−0.40	−0.29	−0.09	−0.24	0.22	0.27	0.62
SD	1.06	1.14	1.10	1.24	1.20			
Height at DXA (cm)								
Mean	120.61	119.56	120.05	118.97	119.27	0.28	0.18	0.12
SD	5.56	5.16	5.35	5.23	5.19			
Height z-score DXA								
Mean	0.11	0.01	0.06	−0.10	−0.04	0.58	0.31	0.32
SD	0.99	1.10	1.04	1.00	1.05			
LBM								
Mean	9.75	9.71	9.73	9.73	9.72	0.10	0.79	0.12
SD	0.14	0.13	0.13	0.13	0.12			
FM								
Mean	8.03	8.02	8.02	8.14	8.08	0.90	0.15	0.55
SD	0.53	0.52	0.52	0.57	0.54			

GA = gestational age and BW = birth weight, no significant difference comparing groups. CA = corrected age, LBM = lean body mass, FM = fat mass, *n* = numbers

The original article has been corrected.

